# A retrospective evaluation of preoperative anemia in patients undergoing radical cystectomy for muscle-invasive urothelial urinary bladder cancer, with or without neoadjuvant chemotherapy

**DOI:** 10.1186/s40064-016-2865-2

**Published:** 2016-07-25

**Authors:** Gustaf Klinga, Amir Sherif

**Affiliations:** Department of Surgical and Perioperative Sciences, Urology and Andrology, Umeå University, 901 85 Umeå, Sweden

**Keywords:** Urinary bladder neoplasms, Neoadjuvant therapy, Cisplatin, Anemia, Cystectomy

## Abstract

**Background and objective:**

Neoadjuvant chemotherapy (NAC) can be associated with anemia, which can lead to more perioperative blood transfusions (PBT). Usage of PBT is associated with worse oncological outcomes. We evaluated the prevalence of preoperative anemia (PA) and the effect on hemoglobin levels depending on surgery timing after NAC.

**Methods:**

A retrospective single-center study with 240 consecutive patients undergoing radical cystectomy (RC) between 2001 and 2014 for muscle-invasive urothelial carcinoma (MIBC). Anemia was defined according to the WHO classification (male ≤ 130 g/L, female ≤ 120 g/L). Multivariable logistical regression was used to identify factors associated with PA and Pearson correlation for evaluating the change in hemoglobin levels depending on surgery timing.

**Results:**

Overall, 128 (53.3 %) patients were anemic pre-RC and 87 (36.3 %) patients received NAC. In a multivariable analysis, age, receipt of NAC, female gender, and low BMI were independent predictors of PA. In patients receiving NAC, the time to surgery from the last NAC cycle was correlated with the change in hemoglobin levels between the initiation of NAC and surgery.

**Conclusions:**

PA was common in patients undergoing RC for MIBC. Receipt of NAC was found to be a strong predictor of PA.

**Clinical message:**

The emerging treatment of cisplatin based neoadjuvant chemotherapy for muscle-invasive bladder cancer, confers an increased risk for preoperative anemia. In the management of this malignancy, preoperative anemia renders further attention and focus.

## Background

Urothelial bladder cancer (UBC) is the fourth most common cancer in the Western World. The majority of patients diagnosed have non-invasive UBC, but around 25 % are muscle-invasive (MIBC) at time of diagnosis (Burger et al. 2013). The recommended treatment for patients with MIBC is radical cystectomy (RC) with lymph node dissection within 3 months to avoid progression and to decrease mortality. Addition of neoadjuvant chemotherapy (NAC) with cisplatin-based combinations is recommended for patients fit for chemotherapy (Witjes et al. 2015). This recommendation is based on the results of prospective randomized trials showing an absolute increase of survival with 5–8 %, 5 years median observation time (Sherif et al. 2004; Vale 2005). RC is considered major surgery and can be associated with significant blood loss and need for preoperative blood transfusions (PBT) (Shabsigh et al. 2009). The use of PBT have been shown to have a negative effect on prognosis in UBC (Abel et al. 2014; Linder et al. 2013; Gierth et al. 2014; Morgan et al. 2013). This detrimental effect has previously also been shown in other cancer types; gastric, colorectal and hepatocellular carcinoma (Ojima et al. 2009; Amato and Pescatori 2006; Wang et al. 2009). The mechanism of this unfavorable outcome is not fully understood. Negative immunomodulatory effects following transfusion have been suggested as one reason (Vamvakas and Blajchman 2007). Difficult surgical conditions in more advanced tumors or in patients with a history of abdominal surgery or radiation therapy of the pelvic organs, may also be a reason for increased use of PBT. Thus adding confounding factors to the long term results following PBT. Preoperative anemia (PA) has also been suggested to be an independent predictor for the outcome following RC. A recent study showed that PA is associated with worse oncological outcomes in patients undergoing RC (Gierth et al. 2015). In order to improve treatment in urothelial MIBC, it is valuable to have patients optimized prior to RC. The use of NAC has postulated side effects such as anemia and this, in combination with a risk of significant blood loss during surgery, can increase the risk for PBT. We sought to evaluate the prevalence of PA, identify associated factors and evaluate the effect of NAC on hemoglobin (Hb) levels in patients with urothelial MIBC undergoing RC.

## Methods

In this retrospective single-center study we analyzed all RCs due to MIBC from 2001 to 2014. From the database we identified 349 consecutive patients, undergoing RC at a tertiary referral center (Norrland university hospital in Umeå, Sweden). We excluded 34 patients with non-urothelial cancer, 71 patients with non-invasive UBC and 4 patients who underwent salvage cystectomy. In summary, 240 consecutive patients constitute the study cohort. The study included 176 (73.3 %) male and 64 (26.7 %) female patients and the median age was 69 years (IQR 62–75). Clinicopathological variables were gathered from patient journals regarding age, gender, BMI (Body Mass Index), ASA (American Society of Anesthesiologists) score, comorbidities (previous heart attack, hypertonia, atrial fibrillation, asthma/Chronic obstructive pulmonary disease (COPD), Diabetes Mellitus), hydronephrosis, estimated blood loss (EBL), receipt of PBT and number of units, receipt of NAC, clinical tumor stage (cT), Hb prior to first NAC-cycle, preoperative Hb and year of surgery.

Information about the change in Hb levels from the initiation of NAC until RC and also time to surgery from last NAC-cycle, was also evaluated. Hydronephrosis was defined as history of hydronephrosis prior to RC. PBT was defined as transfusion of allogenic red blood cells during the operation or in the postoperative hospitalization. The decision to administer blood transfusion was made by treating physicians. No standardized thresholds for transfusion were in place during the study period.

### Statistical analyses

Patients were categorized based on their Hb-level into two groups: Normal Hb-level or anemic. Anemia was defined as Hb ≤ 130 g/L in male and Hb ≤ 120 g/L in female patients, according to the WHO classification (Blanc et al. 1968). Clinical and pathologic characteristics were compared between the groups using Mann–Whitney U test for continuous variables and Fisher’s exact test for categorical variables. Spearman’s rank correlation was used to check the development of EBL and usage of units of PBT over time. Univariable and multivariable logistic regression analysis was performed to identify factors associated with PA. In NAC-patients, Pearson correlation was used to analyze the relationship between the change in Hb-levels following NAC and time to RC. The reported *p* values were two-sided and statistical significance was set at 0.05. All statistical analyses were made using SPSS Statistics^®^ 22 (SPSS, IBM Corp, Armonk, NY, USA).

## Results

### Association of preoperative anemia with clinicopathologic variables

The median Hb was 125.5 g/L (IQR 112.25–138.75). Overall, 128 (53.3 %) patients were anemic while 112 (46.7 %) patients were non-anemic. Anemic patients had an older age (*p* = 0.004), higher ASA-score (*p* = 0.024), a higher incidence of hydronephrosis (*p* = 0.003), received NAC (*p* < 0.001), lower EBL (*p* = 0.035), more PBT (*p* = 0.003), and higher cT (*p* = 0.016), compared to non-anemic patients. There was no significant difference in gender, BMI, history of heart attack, hypertonia, atrial fibrillation, asthma/COPD, Diabetes Mellitus or year of surgery (Table 1). Univariable analyses showed that age (Odds Ratio [OR] 1.046; *p* = 0.004), hydronephrosis (OR 2.564; *p* = 0.003), receipt of NAC (OR 6.744; *p* < 0.001) and cT3 (OR 1.961; *p* = 0.041) or cT4 stage (OR 4.814; *p* = 0.048), both compared to cT2 were associated with PA. ASA III-IV, female gender, history of heart attack, hypertonia, atrial fibrillation, Asthma/COPD, Diabetes Mellitus or BMI were not statistically significant. Multivariable analyses adjusted for the effects of age, gender, BMI, ASA, hydronephrosis, receipt of NAC and cT. Age (OR 1.071; *p* = 0.001) and receipt of NAC (OR 9.668; *p* < 0.001) remained independent predictors of PA in the adjusted model, while hydronephrosis and cT did not. Female gender (OR 0.462; *p* = 0.046) and BMI (OR 0.908; *p* = 0.022) were found to be independent predictors in the adjusted model (Table 2). Further, there was no significant difference in PA comparing complete responders (pT0N0M0) to non-responders or to progressing tumors (*p* = 0.778).

**Table 1:** Patient characteristics of the 240 patients with MIBC treated with radical cystectomy 2001–2014

Parameter	All patients (n = 240)	Non-anemic (n = 112)	Anemic (n = 128)	*p* value
Age (years)	69 (62–75)	68 (61–73)	71 (65–76)	0.004
Sex				0.145
Male	176 (73.3)	77 (68.8)	99 (77.3)	
Female	64 (26.7)	35 (31.2)	29 (22.7)	
BMI	25.5 (22.8–28.4)	25.8 (23.8–28.8)	24.9 (22.5–27.8)	0.054
Missing data	20	10	10	
ASA score				0.024
I	28 (11.8)	19 (17.3)	9 (7.1)	
II	120 (50.6)	57 (51.8)	63 (49.6)	
III	86 (36.3)	32 (29.1)	54 (42.5)	
IV	3 (1.3)	2 (1.8)	1 (0.8)	
Missing data	3	2	1	
Prior heart attack	33 (13.8)	15 (13.4)	18 (14.1)	1
Hypertonia	104 (43.3)	51 (45.5)	53 (41.4)	0.602
Atrial fibrillation	19 (7.9)	8 (7.1)	11 (8.6)	0.812
Asthma/COPD	17 (7.1)	11 (9.8)	6 (4.7)	0.137
Diabetes mellitus	29 (12.1)	13 (11.6)	16 (12.5)	0.845
Hydronephrosis	63 (26.3)	19 (17)	44 (34.4)	0.003
Neoadjuvant chemotherapy	87 (36.3)	17 (15.2)	70 (54.7)	<0.001
EBL (mL)	1000 (700–1712.5)	1200 (725–2000)	1000 (650–1475)	0.035
Missing data	26	15	11	
Units of PBT	4 (2–6)	2 (1–6)	4 (2–6)	0.003
cT				0.017
cT2	178 (72.2)	92 (82.1)	86 (67.2)	
cT3	51 (21.3)	18 (16.1)	33 (25.8)	
cT4	11 (4.6)	2 (1.8)	9 (7.0)	
Year of surgery				0.469
2001–2002	20 (8.3)	12 (10.7)	8 (6.3)	
2003–2005	43 (17.9)	21 (18.8)	22 (17.2)	
2006–2008	55 (22.9)	27 (24.1)	28 (21.9)	
2009–2011	73 (30.4)	34 (30.4)	39 (30.5)	
2012–2014	49 (20.4)	18 (16.1)	31 (24.2)	

Data are shown as median (IQR) or *n* (%)

*BMI* body mass index, *ASA* American Society of Anesthesiologists, *COPD*  chronic obstructive pulmonary disease, *EBL* estimated blood loss, *PBT* perioperative blood transfusion, *cT* clinical tumour stage

**Table 2:** Univariable and multivariable logistic regression analysis of risk factors associated with preoperative anemia

Variable	Univariate			Multivariate		
	OR	95 % CI	*p* value	OR	95 % CI	*p* value
Age	1.046	1.015–1.078	0.004	1.071	1.029–1.114	0.001
Gender						
Male	Referent			Referent		
Female	0.644	0.363–1.146	0.134	0.462	0.216–0.985	0.046
BMI	0.944	0.884–1.007	0.081	0.908	0.837–0.986	0.022
ASA score						
I and II	Referent			Referent		
III and IV	1.708	1.000–2.917	0.050	1.761	0.886–3.500	0.106
Hydronephrosis	2.564	1.388–4.736	0.003	1.761	0.829–3.778	0.140
Reciept of NAC	6.744	3.619–12.569	<0.001	9.668	4.456–20.480	<0.001
Prior heart attack	1.058	0.506–2.213	0.881			
Hypertonia	0.845	0.507–1.410	0.521			
Atrial fibrillation	1.222	0.474–3.155	0.678			
Asthma/COPD	0.452	0.151–1.264	0.130			
Diabetes mellitus	1.088	0.499–2.374	0.832			
cT						
cT2	Referent			Referent		
cT3	1.961	1.029–3.739	0.041	1.781	0.767–4.135	0.179
cT4	4.814	1.011–22.912	0.048	2.878	0.506–16.378	0.233

*OR* odds ratio, *CI* confidence interval, *BMI* body mass index, *ASA* American Society of Anesthesiologists, *NAC* neoadjuvant chemotherapy, *COPD* chronic obstructive pulmonary disease, *cT*  clinical tumor stage

### Development of NAC, anemia, PBT and EBL over time

**Fig. 1:**
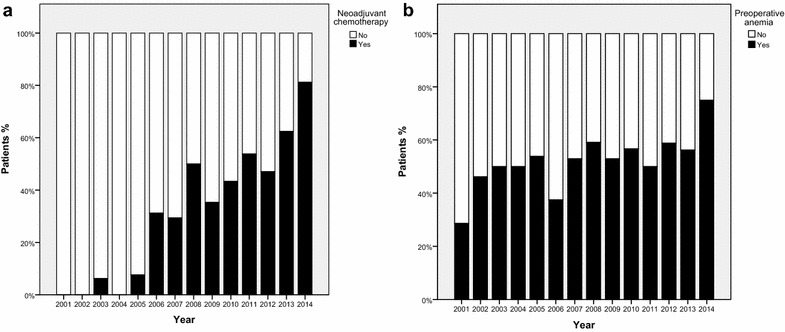
**a** Use of neoadjuvant chemotherapy, 2001–2014. **b** Prevalence of preoperative anemia, 2001–2014. *p* = 0.067

The use of NAC increased during 2001–2014. The first patient receiving NAC was in 2003, with a steady increase during the years. In 2014, 81.3 % of patients with MIBC received NAC at this center (Fig. 1a). There was no statistically significant increase in the prevalence of PA over time (*p* = 0.067; Fig. 1b). Overall, the median EBL was 1000 ml (IQR 700–1712.5). There was no difference in EBL between patients receiving NAC compared to the non-NAC group (*p* = 0.369). The median EBL decreased from 2500 (IQR 2000–11,500) ml in 2001 to 875 (IQR 600–1050) ml in 2014 (*p* = 0.009). The median number of PBT was 4 units (IQR 2–6). No difference in the amount of PBT received was found between patients receiving NAC compared to the no-NAC group (*p* = 0.441). The median number of units transfused perioperatively declined from 7 units (IQR 2–11) in 2001 to 2.5 units (IQR 1–4) in 2014 (*p* = 0.003). Overall, 206 patients (86.2 %) received a PBT during hospitalization for RC.

### Effects of neoadjuvant chemotherapy on hemoglobin levels

**Fig. 2:**
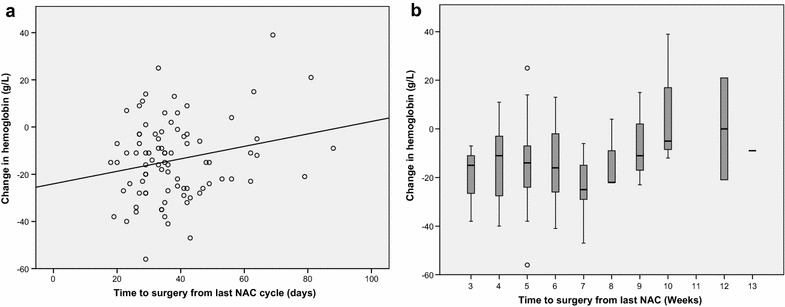
**a** Correlation between time to surgery and change in hemoglobin following NAC. Pearson correlation coefficient: 0.221, *p* = 0.042. **b** Change in hemoglobin following NAC stratified over number of weeks to surgery

Table 3 shows the details of NAC treatment. In NAC-patients (n = 87), the median time to surgery from the last cycle of NAC was 35 days (IQR 29–42). The median change in Hb-level was -15 g/L (IQR −26 to −3.5). The change in Hb-levels can also be expressed in that the number of patients with anemia prior to NAC was 48 (55.2 %), compared to 70 (80.5 %) who had anemia prior to RC post-NAC. The change in Hb following NAC was correlated with time to RC (Pearson correlation coefficient: 0.221, *p* value: 0.042, Fig. 2a). There was an upward trend towards an increasing Hb after 7 weeks (Fig. 2b).

**Table 3:** Information about given NAC and hemoglobin values in the 87 patients who received NAC

Variable	Total (n = 87)
NAC treatment	
MVAC	53 (60.9)
HD-MVAC	21 (24.1)
Cisplatin-Gemzar	6 (6.9)
MVEC	5 (5.7)
Carboplatin-Gemzar	1 (1.1)
MVAC + Taxotere	1 (1.1)
Hemoglobin level (g/L)	
Prior to NAC	127 (117–127)
Prior to surgery	115 (107–123)
Anemic prior to NAC	48 (55.2)
Anemic prior to surgery	70 (80.5)
Number of days from last NAC to surgery	35 (29–42)

Data are shown as median (IQR) or n (%)

## Discussion

The increased risk of adverse outcomes following PBT in conjunction with RC for MIBC, needs to be addressed. Utilization of PBT affects the long term results, not only pertaining to overall and cancer-specific mortality but also to the long term risk of tumor recurrence (7). In general, PA increases the risk for usage of PBT, and we found it of importance to investigate effects of NAC on PA. This with background of optimizing this rapidly emerging treatment, which ultimately aims to improve both overall and cancer-specific mortality. We found that 53.3 % of all the patients had PA. This is a higher prevalence than found in other studies, having reported rates ranging from 39.3 to 51.5 % (Gierth et al. 2014, 2015; Moschini et al. 2015). However, two of the studies excluded patients receiving NAC and in the third study only 2.9 % received NAC. We identified four independent predictors of PA: Age, gender, receipt of NAC and BMI. Receipt of NAC was the strongest predictor (OR 9.668). Anemia has previously been shown to be more common with increasing age (Gierth et al. 2015). A lower BMI could reflect the patient´s state of health, which in turn can have an effect on Hb-levels. The fact that females had a lower risk of PA could partly be explained by a lower anemia threshold in the WHO-definition. Our current praxis is that RC is planned 4 weeks after final NAC-cycle. The planned time to RC in other centers in Sweden varies; some centers proceed after 6–8 weeks. The current EAU-recommendation regarding timing of RC is that the operation is not to be delayed more than 3 months. However, this recommendation is based on single-center studies on chemo naïve patients. A recent study explored the effect of delaying RC > 3 months on survival in a nationwide cohort (Bruins et al. 2016). The investigators also included NAC-patients, showing that the overall survival was similar between patients undergoing RC < 3 months compared to patients with RC > 3 months. This suggests that the 3-month recommendation may not be applicable for NAC-patients. On the other hand, in *NAC*-*non*-*responders*, an increased delay to RC might have a negative effect on survival. The problem with non-responders is currently being handled differently, depending on local traditions. Computerized tomography is commonly used in this center after the second NAC-cycle, for identifying non-responders and in case of non-response or progress, we proceed directly to RC avoiding NAC-cycle 3. Another suggested method is cystoscopy, with a recent study showing that cystoscopy-findings after NAC-cycle 2 are independent predictors of extravesical disease and pathologic downstaging (Mansour et al. 2015). In our study we have seen that utilization of NAC has increased in the last decade. Thus one can assume that with more patients receiving NAC, PA could become more prevalent. Another interesting approach would be to evaluate differences in PA, comparing complete responders to non-responders, thus postulating that micrometastic disease in the latter subgroup might be reflected in PA. Yet in this limited material there was no difference. Further we did not observe any differences in EBL or the amount of PBT received in the NAC group compared to the no-NAC-group. One reason might be that the NAC-patients in our material were significantly younger than the chemo naïve patients (*p* = 0.049). The amount of PBT decreased totally during the studied period. This is probably due to fewer surgeons performing RC following an ongoing national centralization of RC, an increased awareness of both perioperative bleeding and EBL due to national registers (The Swedish bladder cancer register with increasing national coverage from the 2000s and the Swedish cystectomy register which started in 2011) and further the rapidly increasing use of ileal conduit (86.5 % for 2013 in Sweden) instead of other more time consuming urinary diversions.

Within the scope of prospective trials addressing these matters, consideration could be given to identify and treat anemia prior to RC, specifically without allogenic blood transfusions. Another way to address the described problem could be in adjusting the time to surgery after NAC, in order to let the Hb-level recover, and thus reduce the amount of patients with PA and the number of PBT required. In a few patients (n = 12) waiting for surgery more than 7 weeks after final NAC cycle, there was a trend towards a smaller Hb-decrease post-NAC, compared with patients having a shorter interval to RC having a larger decrease. More data is needed to conclusively say if an increased time to surgery could affect the change in Hb-levels. More studies are also required to assess if delayed surgery following NAC has an impact on survival. The limitations of this study include its retrospective design on a relatively small study population. No pathological review was made, and due to the long study period multiple surgeons were involved. Furthermore no data on GFR were available which could add additional information regarding selection of patients to NAC and the individual effect of NAC on renal function. More data from centers where the planned time to RC after NAC is longer could also add value to the analysis of the impact of delayed surgery on PA and the amount of PBT.

## Conclusions

PA was common in patients undergoing RC for MIBC. Receipt of NAC was found to be a strong predictor of PA. This has not been described previously in any evaluations pertaining to NAC-treatment of urothelial MIBC. Strategies to identify and manage patients with PA should be developed to avoid PBT in order to optimize management and prognosis, yet both larger retrospective and also prospective evaluations relating to PA and NAC, need first to be undertaken.

## References

[CR1] Abel EJ, Linder BJ, Bauman TM, Bauer RM, Thompson RH, Thapa P (2014). Perioperative blood transfusion and radical cystectomy: does timing of transfusion affect bladder cancer mortality?. Eur Urol.

[CR2] Amato A, Pescatori M (2006) Perioperative blood transfusions for the recurrence of colorectal cancer. Cochrane Database Syst Rev Jan(1):CD005033 10.1002/14651858.CD005033.pub2 PMC6486137 16437512

[CR3] Blanc B, Finch CAHL (1968). Nutritional anaemias. Report of a WHO scientific group. World Health Organ Tech Rep Ser.

[CR4] Bruins HM, Aben KKH, Arends TJ, van der Heijden AG, Witjes AJ (2016). The effect of the time interval between diagnosis of muscle-invasive bladder cancer and radical cystectomy on staging and survival: a Netherlands Cancer Registry analysis. Urol Oncol.

[CR5] Burger M, Catto JWF, Dalbagni G, Grossman HB, Herr H, Karakiewicz P (2013). Epidemiology and risk factors of urothelial bladder cancer. Eur Urol.

[CR6] Gierth M, Aziz A, Fritsche HM, Burger M, Otto W, Zeman F (2014). The effect of intra- and postoperative allogenic blood transfusion on patients’ survival undergoing radical cystectomy for urothelial carcinoma of the bladder. World J Urol.

[CR7] Gierth M, Mayr R, Aziz A, Krieger S, Wullich B, Pycha A (2015). Preoperative anemia is associated with adverse outcome in patients with urothelial carcinoma of the bladder following radical cystectomy. J Cancer Res Clin Oncol.

[CR8] Linder BJ, Frank I, Cheville JC, Tollefson MK, Thompson RH, Tarrell RF (2013). The impact of perioperative blood transfusion on cancer recurrence and survival following radical cystectomy. Eur Urol.

[CR9] Mansour AM, Soloway MS, Eldefrawy A, Singal R, Joshi S, Manoharan M (2015). Prognostic significance of cystoscopy findings following neoadjuvant chemotherapy for muscle-invasive bladder cancer. Can J Urol.

[CR10] Morgan TM, Barocas DA, Chang SS, Phillips SE, Salem S, Clark PE (2013). The relationship between perioperative blood transfusion and overall mortality in patients undergoing radical cystectomy for bladder cancer. Urol Oncol.

[CR11] Moschini M, Dell’ Oglio P, Capogrosso P, Cucchiara V, Luzzago S, Gandaglia G (2015). Effect of allogeneic intraoperative blood transfusion on survival in patients treated with radical cystectomy for nonmetastatic bladder cancer: results from a single high-volume institution. Clin Genitourin Cancer.

[CR12] Ojima T, Iwahashi M, Nakamori M, Nakamura M, Naka T, Katsuda M (2009). Association of allogeneic blood transfusions and long-term survival of patients with gastric cancer after curative gastrectomy. J Gastrointest Surg.

[CR13] Shabsigh A, Korets R, Vora KC, Brooks CM, Cronin AM, Savage C (2009). Defining early morbidity of radical cystectomy for patients with bladder cancer using a standardized reporting methodology. Eur Urol.

[CR14] Sherif A, Holmberg L, Rintala E, Mestad O, Nilsson J, Nilsson S (2004). Neoadjuvant cisplatinum based combination chemotherapy in patients with invasive bladder cancer: a combined analysis of two Nordic studies. Eur Urol.

[CR15] Vale CL (2005) Neoadjuvant chemotherapy in invasive bladder cancer: update of a systematic review and meta-analysis of individual patient data advanced bladder cancer (ABC) meta-analysis collaboration. Eur Urol 48(2):202–205; discussion 205–206 10.1016/j.eururo.2005.04.006 15939524

[CR16] Vamvakas EC, Blajchman MA (2007). Transfusion-related immunomodulation (TRIM): an update. Blood Rev.

[CR17] Wang C-C, Iyer SG, Low JK, Lin C-Y, Wang S-H, Lu S-N (2009). Perioperative factors affecting long-term outcomes of 473 consecutive patients undergoing hepatectomy for hepatocellular carcinoma. Ann Surg Oncol.

[CR18] Witjes JA, Compérat E, Cowan NC, De Santis M, Gakis G, James N et al (2015) Guidelines on muscle-invasive and metastatic bladder cancer. European Association of Urology 10.1016/j.eururo.2013.11.046 24373477

